# The Impact of PET Imaging on Translational Medicine: Insights from Large-Animal Disease Models

**DOI:** 10.3390/biom15070919

**Published:** 2025-06-23

**Authors:** Zhengyan Deng, Peng Xi, Dongye Zheng, Zhaoheng Xie, Xiangxi Meng, Qiushi Ren

**Affiliations:** 1 Department of Biomedical Engineering, College of Future Technology, Peking University, Beijing 100871, China; 2 National Biomedical Imaging Center, Peking University, Beijing 100871, China; 3 Institute of Biomedical Engineering, Peking University Shenzhen Graduate School, Shenzhen 518055, China; 4 Institute of Medical Technology, Peking University Health Science Center, Peking University, Beijing 100191, China; 5 Key Laboratory of Carcinogenesis and Translational Research (Ministry of Education), Beijing Key Laboratory of Research, Investigation and Evaluation of Radiopharmaceuticals, NMPA Key Laboratory for Research and Evaluation of Radiopharmaceuticals (National Medical Products Administration), Department of Nuclear Medicine, Peking University Cancer Hospital & Institute, Beijing 100142, China

**Keywords:** positron emission tomography, large animals, neurology, infection, metabolic disease, cardiovascular imaging, biomarker discovery

## Abstract

Large-animal models are playing a pivotal role in bridging the translational research gap. Positron emission tomography (PET) imaging is preferred in disease research involving large-animal models. Its ability to non-invasively monitor metabolic activity, receptor–ligand interactions, and pharmacokinetics in real time makes PET imaging an essential tool for evaluating therapeutic efficacy and advancing the development of targeted treatments. This review focuses on recent advancements in dedicated large-animal PET scanners, the utilization of large-animal models for simulating human diseases, and their applications in PET studies. It specifically highlights the critical role of PET imaging in facilitating the development of more effective and safer treatments for infections, chronic heart disease, diabetes, cancer, central nervous system disorders, and addiction, emphasizing its importance in the translational research landscape.

## 1. Introduction

The physiological and anatomical similarities between large animals and humans—particularly in organ size and complexity—make large animals invaluable for modeling human diseases, including complex conditions such as cardiovascular disorders, neurological pathologies, and oncological malignancies. For clinically oriented research, the validation of preliminary findings from rodent studies in large-animal models with high genetic homology to humans is essential [1].Common large-animal models, including pigs (*Sus scrifa*), dogs (*Canis lupus*), and non-human primates (NHPs, such as rhesus monkey *[Macaca mulatta]* and cynomolgus macaque *[Macaca fascicularis]*), are widely employed in translational research. Biomedical imaging in large animals provides non-invasive tools to longitudinally monitor disease progression and therapeutic efficacy. These imaging techniques enable researchers to accelerate the development of safer, more effective diagnostics and therapies for human applications.

Among imaging modalities, positron emission tomography (PET) is particularly prominent in large-animal disease research. Unlike optical imaging, which is commonly used in basic and translational studies in small animals [2], PET imaging distinguishes itself by providing precise quantitative insights into molecular, pharmacological, and metabolic processes at the tissue level. Following the introduction of the first PET scanner [3], researchers conducted experiments on canine subjects to visualize myocardial activity and intracardiac blood distribution. PET’s capacity to non-invasively monitor metabolic activity, receptor–ligand interactions, and pharmacokinetic behavior in real time has since established it as a critical tool for evaluating therapeutic efficacy and advancing targeted treatments. In 2001, a clinical PET scanner integrated with a 16-row computed tomography (CT) system was commercialized as a hybrid PET/CT platform. Subsequently, a small-animal PET scanner with simultaneous magnetic resonance imaging (MRI) capability emerged in 2007, designated as PET/MRI [4].

While conventional human PET scanners may partially meet the demand for dedicated systems in large-animal research, substantial opportunities remain for further improving this equipment. In recent years, a range of dedicated large-animal PET prototypes and commercial scanners with various configurations and architectural designs have been developed, manufactured, and evaluated. These systems bridge the gap between clinical scanners, which are inadequate for imaging specific anatomical structures of large animals, and conventional preclinical scanners with bore sizes too small to accommodate these species.

In this review, we examined the technical aspects of PET scanner design, introduced several state-of-the-art systems for large animals, and explored their applications in large-animal models, such as pigs and NHPs. Additionally, we elucidated tracer developments and methodological innovations that have solidified PET as a cornerstone of molecular imaging.

## 2. The PET Scanner for Large Animals

### 2.1. The Design of the PET Scanner

A traditional PET scanner consists of block detectors arranged in various configurations, typically arranged in circular or polygonal arrays [5]. Each block detector contains a scintillator and a photodetector. The scintillator is composed of crystals, such as bismuth germanate (BGO), lutetium oxyorthosilicate (LSO), lutetium yttrium orthosilicate (LYSO), and lutetium gadolinium oxyorthosilicate (LGSO), among others. These crystals are segmented into arrays of small elements and secured to photodetectors beneath them.

A block detector typically comprises a scintillator segmented into an array of elements with dimensions of approximately 4 mm × 4 mm × 20 mm for clinical systems and 1 mm × 1 mm × 10 mm for preclinical systems, which are read out using single-channel photodetectors [6]. An alternative design is the quadrant-sharing detector, where each scintillator block spans the corners of four photodetectors. This configuration reduces the number of photodetectors required to read out the scintillator elements by nearly 75%.

Photomultiplier tubes (PMTs) and silicon photomultipliers (SiPMs) are commonly used as photodetectors. PMTs were predominant in early PET scanners, whereas SiPMs are now the preferred choice for modern PET systems due to their compact size, magnetic insusceptibility (enabling use in hybrid PET/MRI systems), high photon detection efficiency, and low timing jitter [6].

The depth of interaction (DOI) measurement, which identifies the positron annihilation location within matter, further enables state-of-the-art PET systems, especially for those with a small axial field of view (FOV), like animal PET scanners. Thick detector elements in multi-coincidence-mode systems can degrade the spatial resolution, a phenomenon known as the DOI effect [7]. Scintillation crystals like LSO, LYSO, or gadolinium oxyorthosilicate (GSO) convert gamma-ray energy into optical signals. These signals are captured by PMTs or SiPMs, enabling DOI estimation via the analysis of optical signal arrival time differences across detector layers. DOI measurement is integral to modern PET detector design, as it enhances positron localization accuracy and overall image resolution. However, PET manufacturers vary in their design priorities; some emphasize DOI integration, while others focus on optimizing alternative performance metrics.

### 2.2. Dedicated Large-Animal PET

The transaxial FOV of clinical PET scanners is typically larger than 60 cm, providing high sensitivity and relatively low resolution, whereas small-animal PET scanners usually have transaxial FOVs smaller than 15 cm, offering low sensitivity and relatively high resolution [8,9,10,11,12,13,14]. Consequently, imaging large-animal models with these devices often fails to achieve a satisfactory balance between sensitivity and resolution, necessitating specialized designs tailored to specific species [15,16]. After comprehensive evaluation, a lateral FOV of 20–30 cm is widely accepted for large animals, particularly NHPs, as it balances high resolution and sensitivity for imaging needs [15]. Naidoo-Variawa et al. [17] employed the microPET Focus 220 small-animal scanner (Siemens Preclinical Solutions, Knoxville, TN, USA) to image baboon brains, observing resolution degradation and mismatches in scatter correction algorithms. Notably, its transaxial FOV range aligns with clinical dedicated brain PET systems. However, most major manufacturers have discontinued dedicated high-performance brain PET scanners, and clinical neuroPET studies now predominantly rely on general-purpose devices [18].

UC Davis, Siemens, and other institutions have developed PET/CT systems for large-animal imaging. To date, few dedicated large-animal PET systems exist commercially, with most designed for NHPs [15,16,18,19]. In 2018, the mini-EXPLORER I for NHPs featured a large FOV and PMT photodetectors, but systems for other large animals (e.g., pigs and sheep) remain scarce [20]. In 2019, China’s Institute of High Energy Physics introduced the Eplus-260 primate PET system, utilizing a cerium-doped crystal array and Hamamatsu Photonics (Shizuoka, Japan) photomultiplier tubes [16]. Mediso Ltd. (Budapest, Hungary) and UC Davis later launched high-resolution systems, such as the LFER 150 PET/CT and mini-EXPLORER II, employing LYSO crystals and SiPM photodetectors for veterinary and human brain imaging [15,18]. The LFER 150 is specifically designed for NHP brain imaging, as ^3^ [15]. In 2022, Inviscan SAS (Strasbourg, France) released the IRIS XL-220 PET/CT, a system tailored for NHP imaging with multi-anode PMTs [19]. For larger animals like pigs, researchers often adapt clinical PET scanners due to their size.

**Figure 1:**
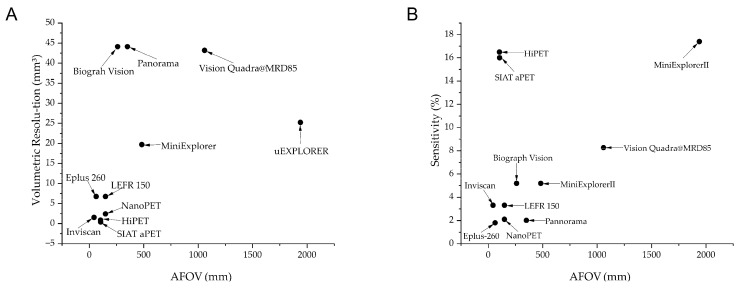
Comparison between different systems. (**A**) Differences in volumetric resolution of different systems; (**B**) differences in sensitivity of different systems. Volumetric resolution refers to the ability of an imaging system to distinguish between two or more objects in three-dimensional space. It is a measure of how well the system can resolve details in all three dimensions: radial (r), tangential (t), and axial (z). Volumetric Resolution (mm^3^) = (Radial × Tangential × Axial).

**Figure 2:**
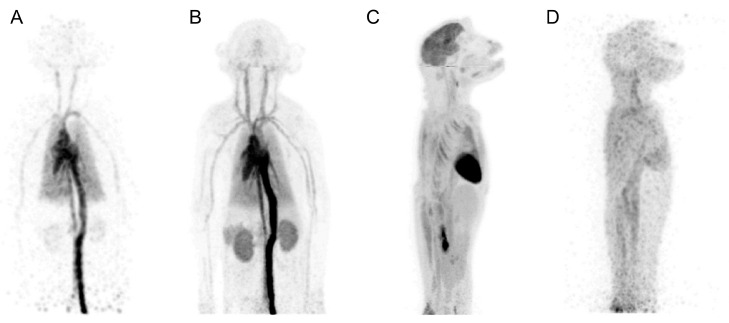
Maximum-intensity-projection images from [^18^F]FDG rhesus monkey study: 1 s frame at 5 s after injection (**A**), 0–30 s after injection (**B**), 55–60 min after injection (**C**), and 18 h after injection (40 min scan) (**D**). This research was originally published in JNM. Eric Berg et al. Development and Evaluation of mini-EXPLORER: A Long Axial Field-of-View PET Scanner for Non-human Primate Imaging. *J Nucl Med*. June 2018, 59(6), 993–998. © SNMMI.

A detailed comparison of these systems alongside other small-animal and clinical PET scanners is provided in Figure 1, indicating their volumetric resolutions and sensitivity against the axial FOV (AFOV). Additional specifications are also provided in Table 1. Representative imaging results from the mini-EXPLORER I are shown in Figure 2.

## 3. Large-Animal Model

### 3.1. Pig Model

Pigs have emerged as a highly relevant animal model across biomedical research, owing to their organ size and weight similarity to humans. Porcine tissues and fluids can be sampled repeatedly with minimal discomfort [21]. The integration of efficient cloning and transgenic technologies, coupled with stable cell lines, position the pigs as an excellent model for scientific research. After nearly half a century of specialized reproduction, large-scale breeding has become feasible, enabling rapid growth cycles and accessibility at a relatively low cost. The versatility of pigs in research is underscored by their widespread applications in studying human developmental processes, congenital diseases, pathogen response mechanisms, xenotransplantation, and vaccine/drug development [22].

In PET imaging, two prominent swine models, the Yucatan pig and the Göttingen pig, are widely adopted, emphasizing the critical role of porcine models in studying complex biological processes. While large-size pig breeds such as Landrace or Yorkshire pose handling challenges during medical procedures, smaller-size models reduce feeding and maintenance costs [21].

The Yucatan pig, or Yucatan miniature pig (YMP), was domesticated from the Yucatan Peninsula of Mexico. YMPs reach an adult body weight of 70–90 kg, aligning with human radiation dose requirements for PET imaging. YMPs are characterized by their gentle temperament, intelligence, disease resistance, and minimal odor [23]. They also exhibit metabolic and pharmacokinetic properties closely resembling humans [24,25], critical for evaluating radiolabeled tracer distribution, metabolism, and excretion in PET.

The Göttingen pig, also known as the Göttingen Minipig (GMP), developed in the 1960s at the University of Göttingen, reaches 12–16 kg at 6 months and 20–27 kg at 12 months. Despite their smaller size, GMPs retain body proportions comparable to standard pigs [26]. Genetic isolation through inbreeding ensures homogeneity and reduced experimental variability [27]. Similar to YMPs, GMPs share human-like physiological, metabolic, and pharmacokinetic traits [28,29], enhancing their utility in PET studies. Both breeds are easily bred, sensitive to environmental conditions, and fed 1–2 times daily. Sows re-enter estrus within 7–10 days post-weaning, enabling rapid reproductive cycles.

Obesity induction in pigs via high-energy diets provides a valuable method for metabolic studies. To model type 1 diabetes, β-cell dysfunction is induced using streptozotocin (STZ), generating hyperglycemic phenotypes [30]. STZ-induced diabetic pigs exhibit metabolic changes in carbohydrate, fat, and amino acid metabolism, mirroring those in humans with untreated diabetes [31]. The porcine pancreas and islets also share functional similarities with humans [32]. In contrast, rodent models fail to fully replicate phenotypes such as insulin resistance, obesity, and hypercholesterolemia, which contribute to glucose intolerance and type 2 diabetes [33]. Thus, STZ-induced and diet-induced diabetic pigs serve as robust large-animal models for diabetes research.

As animal models approach human body or heart weight, differences diminish, establishing pigs as a preferred model for chronic heart disease (CHD) studies [1,34]. Their coronary arterial anatomy and ischemic tolerance similarities to humans further suit CHD pathophysiological research [35]. However, inducing large myocardial infarction with global left ventricular dysfunction remains challenging, as pigs often experience fatal arrhythmias and low ischemia tolerance [36]. Genetically engineered pigs have demonstrated their potential in elucidating human disease mechanisms (Table 2), solidifying PET imaging as a key tool for dissecting disease pathways.

### 3.2. Non-Human Primate Model

The NHP model is indispensable in human disease research due to its genetic similarity to humans and comparable drug metabolism and pharmacokinetics [15,42]. NHPs also exhibit physiological congruence with humans in organ structure, immune responses, metabolism, and neurobiology. Their advanced cognitive abilities and complex behaviors further distinguish them as ideal models for studying neurological, cognitive, and behavioral disorders. Consequently, NHPs are widely used in infectious diseases, central nervous system (CNS) diseases, and substance abuse research [15,42]. For instance, in tuberculosis studies, NHPs replicate the full spectrum of human Mycobacterium tuberculosis (Mtb) infection, including latent tuberculosis—a feature that is absent in mouse models, which fail to form human-like granulomas [43,44,45].

While small-animal models dominate CNS research, their limitations are obvious. For example, rodents exhibit higher absolute expression of the drug efflux transporter P-glycoprotein in the brain compared to humans, pigs, and NHPs, limiting their relevance for certain CNS studies [46]. PET studies confirm divergent P-glycoprotein activity across species [47,48]. Additionally, primate Betz/extratelencephalic neurons display unique gene expression and electrophysiological features—such as pauses, bursting, and spike-frequency acceleration—observed in NHPs but absent in rodents [49]. In a Parkinson’s disease (PD) study, 1-Methyl-4-phenyl-1,2,3,6-tetrahydropyridine (MPTP)-induced animal models are common, yet they fail to replicate human PD hallmarks, like Lewy bodies [50]. MPTP-induced mouse models exhibit strain- and age-dependent responses to MPTP, with acute injury and rapid recovery complicating long-term therapeutic evaluation [51]. In contrast, MPTP-induced NHP models recapitulate both motor and non-motor PD symptoms, solidifying their superiority for PD studies [52].

The genetic overlap between humans and NHPs has spurred widespread use of genetically engineered NHP models and gene therapies (Table 3), particularly in CNS research. Alongside genetic tools, toxin-induced models are employed to study CNS disorders [53]. NHPs also remain irreplaceable in infectious disease research (Table 4), enabling the investigation of single- and multi-pathogen interactions [54].

Beyond infectious diseases, the cognitive capacities and complex behaviors of NHPs provide a critical platform for studying drug effects on the brain and substance abuse mechanisms. Although rodent models are widely used in addiction research, key physiological and behavioral differences persist between rodents and primates. For example, opioid receptor expression and localization in the rodent CNS differ significantly from primates, with δ-opioid binding sites in the rodent spinal cord being far more extensive than in primates. This makes it likely that the pharmacological effects of such drugs are more numerous than in the primate spinal cord, especially in humans [59]. Furthermore, NHPs’ capacity for higher-order cognitive tasks offers unique insights into psychiatric and psychological impairments in human drug users [60].

To support these investigations, PET imaging serves as a pivotal tool. By capturing in vivo metabolic processes and integrating with MRI or CT, PET enables researchers to dissect the complex interactions of genetics, toxins, and drug effects on the NHP CNS (Table 4).

**Table 4:** Infectious disease NHP models.

Pathogen	Phenotype	Potential Applications and Significance
Simian immunodeficiency virus (SIV) [61]	High levels of virus replication associated with a high magnitude of cytokine/chemokine response	Difference between progressive and non-progressive disease courses
Simian–human immunodeficiency virus (SHIV) [62]	Lower viral burden and viral control during cART; exhibited less peripheral CD4 depletion and lower gut immune dysfunction and immune activation	SHIV macaque model may be better for identifying initial vaccine candidates
Sudan virus [63]	Early stage: viremia, granulocytosis, lymphopenia, albuminemia, thrombocytopenia, and decreased expression of HLA-class transcripts; mid-to-late stage: fever and petechial rashes, high levels of pro-inflammatory mediators and pro-thrombotic factors; end stage: shock and multi-organ failure	The development of vaccines and therapeutics
Mycobacterium tuberculosis (Mtb) [64]	Complement C1q increased after Mtb infection; C1q increased after Bacillus Calmette Guérin (BCG) vaccination	C1q can serve as a marker of progressive TB disease
Severe acute respiratory syndrome–coronavirus 2 (SARS-CoV-2) [65,66]	Increased levels of monocytes and chemokines; interstitial macrophages accumulate in the lungs	The immune events of the host response and viral replication and disease progression; therapeutic strategies
	Both intranasal and intragastric inoculation with SARS-CoV-2 caused pneumonia and GI dysfunction	Inflammatory cytokines are possible connections for the pathogenesis of SARS-CoV-2 between the respiratory and digestive systems

### 3.3. Sheep Model

Sheep are a widely used model in reproductive research due to their anatomical similarity to humans, including comparable fetus-to-pelvis ratios and spontaneous pelvic organ prolapse [67]. Their accessibility, affordability, and docility further enhance their practicality [68]. In CNS studies, sheep brains demonstrate high structural homology with humans [69]. Critically, their neurodegenerative processes recapitulate human pathology, making them invaluable for studying disorders like Huntington’s disease (HD) [70]. Similarities in spinal anatomy and cerebrospinal fluid (CSF) volume further reinforce their translational relevance [71]. Snell et al. [72,73] have extensively developed sheep CNS disease models, most notably the OVT73 HD model. This transgenic line, generated via DNA injection into zygotes, carries CAG repeats within the human disease-causing range—unlike rodent models with artificially elongated repeats [70,72,74]. OVT73 sheep live up to 10 years, enabling longitudinal studies that track HD progression and accelerate therapeutic development. When integrated with PET imaging, OVT73 provides a powerful platform for HD research.

## 4. Application

### 4.1. Infection

**Figure 3:**
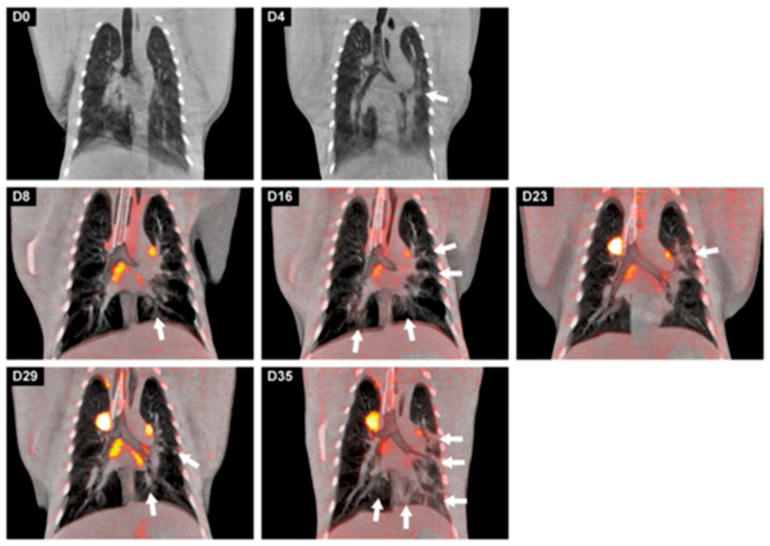
Longitudinal development of both lung lesions and metabolic activity in tracheobronchial lymph nodes of a cynomolgus macaque over time after a SARS-CoV-2 infection. Representative coronal slices with a thickness of 3 mm of the cynomolgus macaque were used for visualization. On days 0 and 4, only CT images were obtained; afterwards, until day 35, CT was combined with PET (LFER 150). The location of the lesions, marked with arrows, differed almost per time point but are most prominently localized in the left lung on day 35. This research was originally published in *Viruses*. Böszörményi, K.P. et al., The Post-Acute Phase of SARS-CoV-2 Infection in Two Macaque Species Is Associated with Signs of Ongoing Virus Replication and Pathology in Pulmonary and Extrapulmonary Tissues. *Viruses*, 2021. 13(8).

Respiratory infections have long been a major research focus. The coronavirus disease 2019 (COVID-19) pandemic has profoundly influenced PET research. During the initial stages of the pandemic, the limited understanding of SARS-CoV-2 necessitated animal models to study infection pathology and develop therapeutics and vaccines. In NHP models, infected NHPs exhibit varied COVID-19 progression, making them valuable for investigating infection pathology, therapeutics, and vaccines [75,76,77,78]. However, these findings were predominantly obtained through invasive methods, underscoring the critical need for non-invasive tools to visualize infection and treatment responses. PET imaging, with its high resolution and non-invasive nature, emerged as a complementary tool for monitoring NHPs. The advent of PET technology has revolutionized the ability to study respiratory infections, offering a powerful method to explore infection outcomes without invasive procedures (Figure 3). Using [^18^F]FDG PET imaging, researchers gain insights into the molecular-level onset, progression, and spread of COVID-19-induced inflammation in the lungs and nervous system [79,80].

Prior to COVID-19, the scientific community focused on Mtb, a respiratory disease that has afflicted humanity for millennia. Given its inflammatory pathogenesis, [^18^F]FDG is well suited for imaging tuberculosis. Decades of research have revealed intricate mechanisms underlying lymph node and granuloma dynamics [81,82,83], findings with significant implications for immunization strategies in vulnerable populations. By elucidating complex tuberculosis mechanisms, PET technology advances interventions against this persistent disease.

Human immunodeficiency virus (HIV) is a key focus in large-animal studies. While humanized mouse models allow for direct HIV infection and provide insights into antiretroviral therapy efficacy, they cannot fully recapitulate HIV-1 pathogenesis [82]. However, HIV is unable to infect NHPs in the wild, leading to the discovery of simian immunodeficiency virus (SIV) in Africa. Although SIV shares similarity with HIV-1, it lacks key structural features and is non-pathogenic in most natural hosts despite high viral replication levels [84]. Nevertheless, SIV offers insights into HIV mechanisms, such as significant enteric virome expansion linked to SIV-induced immunopathogenesis [85,86]. Additionally, simian–human immunodeficiency virus (SHIV), a chimeric virus combining SIV and HIV genetic elements, was developed to further study HIV.

In HIV vaccine research, both the virion and the subunit vaccine entities exhibit an extended biodistribution profile, and their in vivo distribution is more compatible with ^64^Cu [87,88]. Research on SIV and SHIV, combined with PET imaging, provides critical tools for studying HIV pathology, vaccines, therapies, and immune responses, accelerating progress toward prevention and treatment strategies. PET imaging in large animals facilitates vaccine development, evaluation of novel drug treatments, and testing of new tracers.

### 4.2. Chronic Heart Disease

PET imaging stands as an invaluable asset in the diagnosis and monitoring of CHD, providing a comprehensive evaluation of cardiac function. Central to this application is myocardial perfusion imaging (MPI) [89,90], which quantifies relative differences in myocardial blood flow distribution under rest and stress conditions (induced via exercise or pharmacological agents). Myocardial arterioles distal to significant coronary stenosis dilate via autoregulation to maintain resting blood flow. During stress, normal vascular beds undergo significant vasodilation, whereas stenotic regions exhibit limited dilation, resulting in perfusion disparities that manifest as "defects" in MPI. Large-animal studies commonly model CHD through artificially induced coronary artery occlusion [90].

MPI primarily assesses coronary artery disease by evaluating myocardial blood flow. Key radiotracers include [^15^O]H_2_O, a metabolically inert, freely diffusible tracer for myocardial viability assessment [91,92,93], and [^13^N]NH_3_, whose uptake directly reflects blood flow [90,94]. In large-animal studies, MPI is often integrated with [^18^F]FDG to simultaneously assess tissue viability [36]. Researchers have also tested novel tracers in a permanent occlusion model and occlusion–reperfusion model, demonstrating efficacy in these systems [90,94,95]. Collectively, PET imaging has significantly advanced CHD research, providing critical insights to guide therapeutic innovation and refine clinical management.

### 4.3. Diabetes

The loss of functional β-cell mass (BCM) is a hallmark feature of both type 1 and type 2 diabetes (T1D and T2D). The current understanding of progressive BCM changes during diabetes pathogenesis relies on post-mortem biopsies [96]. Notably, BCM declines before T2D onset, and emerging evidence suggests that impaired glucose tolerance—a precursor to T2D—is linked to reduced BCM [97,98,99]. Hypotheses proposed that T2D progression involves reduced β-cell regeneration or accelerated β-cell loss [100]. PET imaging offers a non-invasive method to visualize BCM, allowing for high-resolution quantification of pancreatic β-cells in diabetes research [96].

Beyond BCM assessment, glucagon-like peptide-1 (GLP-1) has emerged as a critical focus in diabetes studies. GLP-1 is produced in the gut, brainstem, and endocrine pancreas, exerting energy balance regulation via the GLP-1 receptor (GLP-1R). Its primary physiological role is insulinotropic action, accompanied by the suppression of glucagon release, mediated through somatostatin from pancreatic δ cells. GLP-1 also exhibits glucose-lowering properties, including CNS-mediated satiety, blood pressure reduction, and modulation of postprandial lipid metabolism [99]. Advances in GLP-1R-targeted tracers have enabled PET imaging to elucidate the signaling dynamics of GLP-1, offering insights into BCM and therapeutic development [32,101]. Collectively, PET imaging holds revolutionary potential for diabetes research, linking mechanistic understanding with innovative diagnostic and therapeutic strategies.

### 4.4. Cancer

PET imaging is a tool in cancer research involving large animals, offering detailed insights into biochemical processes within tumors. This capability significantly enhances cancer diagnosis, staging, monitoring, and treatment. The radiotracer [^18^F]FDG, widely used in species (including humans, large animals, and small animals), capitalizes on elevated glucose uptake driven by rapid tumor cell proliferation. Similarly, [^68^Ga]Ga-DOTATATE, targeting somatostatin receptor type 2 overexpression, is a cornerstone in neuroendocrine tumor diagnosis and management [102]. Currently, researchers focus on developing new radiotracers and testing them in large-animal models [103,104,105,106]. Although the use of large-animal models in oncology has declined in recent years due to advances in humanized mice, which offer a cost-effective platform for studying tumor biology, including the human immune microenvironment [107], these models retain relevance in translational medicine, particularly in drug development, owing to their anatomical and physiological similarity to humans [108,109]. Moreover, large-animal models play a pivotal role in evaluating cancer therapeutics, as PET imaging enables real-time monitoring of treatment progression and efficacy, accelerating the development of innovative diagnostic and therapeutic strategies [110].

### 4.5. Central Nervous System Disease

#### 4.5.1. α-Synucleinopathies

PET imaging has proven instrumental in investigating α-synuclein (α-syn) pathologies, including PD and Lewy body disease (LBD) [111]. These disorders are characterized by the misfolding and abnormal aggregation of pathological α-syn in neurons, eventually forming insoluble α-syn inclusion bodies (the primary component of Lewy bodies) [112]. These aggregates propagate across the central and peripheral nervous systems, causing diverse motor and non-motor symptoms. Diagnosing α-synucleinopathies remains challenging, relying on clinical history, signs, and symptoms; accurate diagnosis necessitates multidisciplinary collaboration [111]. The radiotracer [^18^F]F-dihydroxyphenylalanine ([^18^F]F-DOPA), a marker of monoaminergic nerve terminal function, has been extensively used to evaluate the severity and progression of presynaptic nigrostriatal dysfunction in PD, as striatal [^18^F]F-DOPA uptake reflects aromatic amino acid decarboxylase activity [113]. Similarly, [^18^F]FDG has revealed distinct metabolic declines in glucose utilization in NHP brains affected by α-synucleinopathies [114,115]. MPTP-induced PD models in large animals have further delineated presymptomatic and symptomatic phases of nigrostriatal neurodegeneration [53,116,117]. Together, PET imaging provides an indispensable foundation for elucidating α-synucleinopathy pathophysiology and advancing therapeutic strategies.

#### 4.5.2. Alzheimer’s Disease

Alzheimer’s disease (AD) is a progressive neurodegenerative disorder characterized by cognitive decline, memory impairment, and the loss of functional independence. Despite extensive research, its pathogenesis remains incompletely understood. The predominant hypothesis posits that dysregulated amyloid-β (Aβ) production and clearance triggers neuronal degeneration and cognitive decline [118]. Aβ plaques disrupt interneuronal communication and contribute to neurodegeneration. Another key pathological feature involves neurofibrillary tangles—tau (τ) protein aggregates within neurons—which directly impair neuronal microtubular transport [119]. To unravel AD pathology, researchers have advanced PET imaging techniques. The ^11^C-labeled Pittsburgh compound B ([^11^C] PIB), a radiotracer specifically designed to bind to β-amyloid plaques, enables detection of early AD pathological processes and subsequent neurodegeneration [120,121]. In the field of AD research in large animals, a current prominent research direction involves exploring novel radiotracers in large-animal models to detect Aβ deposition and abnormal τ protein aggregation and facilitate new drug development, particularly in NHP models [122,123,124,125,126]. These advancements underscore PET’s pivotal role in deciphering AD mechanisms and accelerating therapeutic and diagnostic innovations. By leveraging these novel radiotracers, studies in NHPs have refined our understanding of AD pathology, paving the way to targeted interventions.

#### 4.5.3. Huntington’s Disease

HD is a rare inherited neurological disorder characterized by progressive motor dysfunction, cognitive decline, and emotional dysregulation. The disorder stems from a mutation in the huntingtin (HTT) gene, involving an abnormal expansion of cytosine–adenine–guanine (CAG) repeats in exon 1, which leads to the production of toxic mutant HTT protein [127]. This aberrant protein accumulates as neuronal aggregates, disrupting cellular homeostasis and inducing neurodegeneration, particularly within the cerebral cortex and striatum, driving progressive motor and cognitive deterioration.

**Figure 4:**
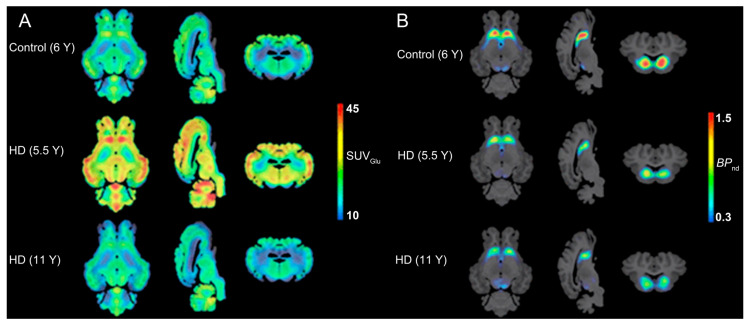
PET examination on HD sheep. (**A**) Average glucose uptake images showing the coronal, sagittal, and axial views in each group (*n* = 3/group). The averaged PET images are overlaid onto the MRI template for anatomical reference. (**B**) Imaging dopamine metabolism using [^18^F]FDOPA. Tracer uptake assessment and averaged [^18^F]FDOPA parametric maps for control, 5.5 Y, and 11 Y HD sheep (*n* = 3/group) are overlaid onto the T1w MRI template for anatomical reference. Reprinted (adapted) with permission from Williams, G. K., Akkermans, J., Lawson, M., Syta, P., Staelens, S., Adhikari, M. H., Morton, A. J., Nitzsche, B., Boltze, J., Christou, C., Bertoglio, D., & Ahamed, M. (2024). Imaging Glucose Metabolism and Dopaminergic Dysfunction in Sheep (*Ovis aries*) Brain Using Positron Emission Tomography Imaging Reveals Abnormalities in OVT73 Huntington’s Disease Sheep. *ACS chemical neuroscience*, 15(21), 4082–4091. https://doi.org/10.1021/acschemneuro.4c00561. Copyright 2025 American Chemical Society.

Current diagnostic frameworks lack standardized criteria for diagnosing HD prior to clinical onset, relying primarily on motor symptom assessment [128]. Williams et al. [129] demonstrated the feasibility of PET imaging in HD sheep using [^18^F]FDG and [^18^F]FDOPA to map cerebral glucose metabolism and dopaminergic activity, establishing a translational tool for preclinical research (Figure 4). NHPs are similarly important models, with novel radiotracers developed to probe HD pathophysiology [130,131,132,133]. In summary, preclinical large-animal studies are rapidly advancing, offering transformative insights for clinical diagnostics and therapeutics.

#### 4.5.4. Psychiatric Disorders

In neuroscience, PET has emerged as an indispensable tool, not only in investigating α-synucleinopathies, AD, and HD but also in exploring psychiatric disorders, such as schizophrenia, stress-related disorders, anxiety, and depression. These conditions, each with distinct pathogenic mechanisms, pose significant challenges to developing effective diagnostics and therapies. Smucny et al. [134] investigated the link between maternal immune activation during pregnancy and schizophrenia risk in offspring, employing PET with the radiotracer [^18^F]fluoro-l-m-tyrosine (an L-DOPA analog) in rhesus monkeys. Their findings revealed that maternal immune activation increases susceptibility to schizophrenia-related outcomes. Wokeford et al. [135] explored the influence of gender and social status on the efficacy of serotonergic drugs using the radiotracer [^18^F]MPPF (4-(2′-methoxyphenyl)-1-[2′-(N-2″-pyridinyl)-p-fluorobenzamido]ethylpiperazine). They demonstrated that these factors significantly modulate treatment responses. Furthermore, PET studies in NHPs have uncovered associations between childhood anxious temperament and increased risks for anxiety, depression, and substance abuse, providing mechanistic insights for refining interventions [136,137,138]. Together, PET imaging in NHPs provides a versatile platform to elucidate the neurobiological underpinnings of psychiatric disorders. These advances highlight PET’s ability to illuminate pathogenic pathways and accelerate translational breakthroughs in neuroscience.

### 4.6. Addiction

Addiction refers to a chronic, recurring disorder characterized by an inability to control the pursuit, continued use, or compulsive behaviors despite harmful consequences. It is typically associated with physical or psychological dependence on a substance or behavior. Ethical constraints governing human addiction studies necessitate reliance on observational methodologies that limit systematic control of variables, particularly for invasive experimental protocols and genetic heterogeneity among subjects. Animal models, despite their inability to replicate the psychosocial complexities of substance abuse, provide critical experimental control over pharmacokinetic parameters and enable longitudinal monitoring of neurocognitive adaptations through controlled dosing regimens [139]. These models have proven invaluable for elucidating neurobiological mechanisms, such as reward-related learning. Central to this pathology is dysregulation of the mesolimbic dopamine system, which includes dopaminergic projections from the ventral tegmental area to the ventral striatum (nucleus accumbens) and cortical regions [140].

PET studies have consistently shown that long-term cocaine use on NHPs is associated with reduced dopamine D_2_/D_3_ receptor (D_2_/D_3_R) availability [141,142]. A study conducted by Allen et al. [143] on female cynomolgus monkeys extended the previous research findings based on male monkeys to females, indicating gender differences in the availability of D_2_/D_3_R related to vulnerability and long-term cocaine use. The study utilized [^11^C]raclopride (a D_2_R selective antagonist) to quantify dopamine D_2_R availability during addictive drug exposure and [^18^F]FECNT (a novel tracer targeting the dopamine transporter) to assess dopamine transporter availability [143]. Furthermore, studies using [^11^C]raclopride and its analog [^18^F]fluoroclebopride on NHPs have demonstrated nicotine-induced dopamine release in reward-related circuits [144,145]. Together, these findings underscore PET imaging’s unique capacity to unravel neurochemical dynamics in addiction, from substance-induced receptor adaptation to withdrawal pathophysiology. Such insights are pivotal for advancing targeted therapeutic strategies.

In summary, a summary of radiotracers used in large-animal models is listed in Table 5.

**Table 5:** Summary of radiotracers used in large-animal models.

Tracer	Application	Mechanism	Reference
[^11^C]acetate	Myocardial perfusion imaging (MPI)	[^11^C]acetate is rapidly metabolized in cardiomyocytes to acetyl-coenzyme A; acetyl-CoA is a key intermediate in fatty acid oxidation and glucose metabolism in cardiomyocytes	[93]
[^11^C]dihydrotetrabenazine ([^11^C]DTBZ)	Parkinson’s disease (PD)	[^11^C]DTBZ binds to the VMAT2 of synaptic vesicles in monoaminergic neurons, which reflects the density and functional state of VMAT2	[116,117]
^11^C-labeled Pittsburgh compound B ([^11^C]PIB)	Alzheimer’s disease (AD)	[^11^C]PIB is specifically designed to bind to β-amyloid plaques in the brain	[120,121]
[^11^C]raclopride	Addiction	D_2_R sensitivity to reward decreases in addiction; [^11^C]raclopride can Quantify dopamine D_2_R availability in the brain	[141,144]
[^11^C]AZ12204657 and [^11^C]MK-7246	Diabetes	Target to GPR44 to β-cell imaging	[96]
[N-methyl-^11^C]-cholylsarcosine ([^11^C]CSar)	Cancer	[^11^C]CSar can assess bile acid excretion in the liver	[110]
[^11^C]MPC-6827	AD	MPC-6827 is a microtubule-targeting agent that binds to tubulin sites with high affinity	[122]
[^11^C]GSK215083	AD	[^11^C]GSK215083 is a selective 5-HT_6_ tracer	[124]
[^11^C]BIO-1819578	AD	BIO-1819578 is an O-GlcNAcase PET ligand that is an enzyme associated with the development of τ	[126]
[^11^C]PHNO	Addiction (nicotine)	[^11^C]PHNO has a higher affinity for D_3_ vs. D_2_ DA receptors, which allows for regional interpretation of D_3_ and D_2_ receptors	[144]
[^11^C]CHDI-180R, [^11^C]CHDI-626 and [^11^C]CHDI-650	Huntington disease (HD)	[^11^C]CHDI-180R, [^11^C]CHDI-626, and [^11^C]CHDI-650 are mHTT aggregate-specific PET ligands	[131,132,133]
[^13^N]NH_3_	MPI	[^13^N]NH_3_ can be taken up by cardiomyocytes via an amino acid transport system, and its uptake is proportional to myocardial blood flow	[90,94]
[^15^O]H_2_O	MPI	[^15^O]H_2_O is a freely diffusible and metabolically inert tracer; can be used to establish myocardial blood flow	[91,92,93]
[^18^F]FDG	Cancer, tuberculosis, COVID-19 infection, and psychiatric disorders	Increased glucose metabolism in tumor tissue or inflammation	[79,81,82,83,136,138]
	Myocardial viability imaging, AD, HD, and α-syn disease	Decreased glucose metabolism in damaged cardiomyocytes, AD brain, or α-syn disease brain	[36,114,115,116,129]
[^18^F]F-DOPA	PD	Striatal uptake of [^18^F]F-DOPA reflects aromatic amino acid decarboxylase activity	[113]
[^18^F]Flurpiridaz	MPI	[^18^F]Flurpiridaz works by binding to mitochondrial complex 1 in the heart	[94,95]
[^18^F]KS1	Cancer	[^18^F]KS1 is a fluoroethoxy furanose ring-containing ascorbate derivative, to track ROS in prostate tumor	[103]
[^18^F]TTDP	Cancer	Target to T cell immunoglobulin and ITIM domain	[105]
[^18^F]BMS-986229	Cancer	A ^18^F-labeled macrocyclic peptide-based PET ligand for imaging PD-L1	[106]
[^18^F]fluoro-2-deoxy-D-galactose ([^18^F]FDGal)	Cancer	[^18^F]FDGal can assess galactose metabolism	[110]
[^18^F]fluoro-l-m-tyrosine	Schizophrenia	L-m-tyrosine is an analogue of L-DOPA	[134]
[^18^F]**1**	HD	An mHTT aggregate-specific PET ligand	[130]
[^18^F]**3**	AD	A BACE1 PET ligand	[125]
[^18^F]**92**	AD	A new tracer that is designed to combined Aβ	[123]
[^18^F]MPPF (4-(2′-methoxyphenyl)-1-[2′-(N-2′′-pyridinyl)-p-fluorobenzamido]ethylpiperazine)	Psychiatric disorders	A radioligand employed for imaging 5-HT_1A_ receptors	[135]
[^18^F]FECNT	Addiction (cocaine)	Target to dopamine transporter	[141]
[^18^F]fluoroclebopride	Addiction (cocaine)	An ^18^F-labeled raclopride	[142]
[^18^F]Fallypride	Psychiatric disorders and addiction (nicotine)	A high-affinity dopamine D_2_/D_3_ receptor antagonist	[137,145]
^64^Cu labeled to Photoactivatable-Green Fluorescent Protein-HIV-BaL	HIV infection	PET/CT and fluorescent microscopy dual-modal imaging	[87]
^64^Cu labeled to vaccine-loaded nanoparticle	HIV infection	A combination of PET and fluorescence imaging	[88]
[^68^Ga]Ga-DOTATATE	Cancer	Based on somatostatin receptor (SSTR) type 2	[102]
[^68^Ga]Ga-DO3A-VS-Cys(40)-Exendin-4	Diabetes	Target to GLP1-R	[32,101]
[^89^Zr]hu5A10	Cancer	A humanized uncomplexed and catalytically active prostate-specific antigen-targeting IgG_1_-mAb	[104]

## 5. Conclusions

PET has evolved into an indispensable tool for investigating physiological and pathological processes in large animals. The integration of advanced PET technology, tailored disease models, and diverse applications establishes large-animal PET studies as pivotal bridges between preclinical research and clinical outcomes. Due to the closer physiological and anatomical resemblance of large-animal models to humans, molecular probes and drug mechanisms tested in these models exhibit higher predictive value, enhancing their utility in translational medicine. By labeling radioisotopes onto drug candidates, PET dynamically tracks their distribution in organs and lesions. PET further enables the evaluation of pharmacodynamic efficacy through metabolic monitoring in large animals. Future advancements in large-animal PET imaging will likely focus on developing dedicated systems with higher resolution, sensitivity, and specificity, or designing tri-modal/multimodal imaging platforms to acquire multidimensional data. As methodologies and insights expand, PET’s integration into large-animal studies promises to drive transformative breakthroughs in basic research and clinical medicine.

## Figures and Tables

**Table 1:** The performance of representative small-animal and large-animal and clinical PET scanners.

		Name of Scanner	Crystal Size (mm^3^)	Transaxial/Axial Field of View (mm)	Volumetric Resolution (mm^3^)/(Radial × Tangential × Axial)	Reconstruction Method	Sensitivity (%)	Energy Window (keV)	Coincidence Window (ns)	Time-of-Flight Resolution (ps)	Depth of Interaction Information
Animal PET Scanner	Small-animal PET	NanoPET [12]	1.12 × 1.12 × 13	123/9.4~150	2.40 (1.49 × 1.39 × 1.16)	Filtered Back Projection (FBP)	2.1	250–750	5	--	No
		HiPET [13]	1.01 × 1.01 × 6.1 & 1.55 × 1.55 × 8.9	131/104	0.882 (1.0 × 0.98 × 0.9)	FBP	16.5	150–750	6		Yes
		SIAT aPET [14]	1.0 × 1.0 × 20	<111/105.6	0.37 (0.68 × 0.8 × 0.69)	Ordered Subset Expectation Maximization	16	250–750	--	--	Yes
	Large-animal PET	Inviscan [19]	1.6 × 1.6 × 16	170/45	1.53 (1.05 × 0.97 × 1.51)	FBP	3.3	250–750	5.2	--	Yes
		LFER 150 [15]	1.51 × 1.51 × 10	200/150	6.73 (2 × 1.86 × 1.81)	FBP	3.3	400–600	5	--	No
		Eplus-260 [16]	1.9 × 1.9 × 10	190/64	6.72 (2.4 × 2 × 1.4)	FBP	1.8	460–660	2	--	No
		MiniExplorer II [18]	2.76 × 2.76 × 18	520/483	19.69 (2.62 × 2.88 × 2.61) @1/2 AFOV	FBP	5.18	--	--	409	No
Clinical PET Scanner	Short-axial FOV	Panorama [8]	2.76 × 2.76 × 18.1	760/351	44.1 (2.72 × 3.02 × 2.74)	FBP	2.01	--	--	189	No
		Biograph Vison [9]	3.2 × 3.2 × 20	780/261	44.1 (3.5 × 3.6 × 3.5)	FBP	5.18	--	--	203	No
	Long-axial FOV	Vision Quadra@MRD85 [10]	3.2 × 3.2 × 20	780/1060	43.16 (3.19 × 3.58 × 3.78)	FBP	8.26	--	--	228	No
		uExplorer [11]	2.76 × 2.76 × 18.1	760/1940	25.2 (3.0 × 3.0 × 2.8)	FBP	17.4	--	--	500	No

The resolution and sensitivity values obtained at the axial center of the scanners. FWHM was used to calculate the volumetric resolution of the point source (10 mm radial offset), which is the product of the resolution in the axial, tangential, and axial directions. The reconstruction method refers to the reconstruction algorithm for the test of the resolution. ^4^ Volumetric resolution refers to the ability of an imaging system to distinguish between two or more objects in three-dimensional space. It is a measure of how well the system can resolve details in all three dimensions: radial (r), tangential (t), and axial (z). Volumetric Resolution (mm^3^) = (Radial × Tangential × Axial).

**Table 2:** Genetically engineered pig models.

Disease	Genetic Modification	Phenotype	Potential Applications
Diabetes [37,38]	Knockin dominant-negative glucose-dependent insulinotropic polypeptide (GIP) receptor	Reduced insulinotropic effect of GIP; reduced glucose tolerance and insulin secretion	Development and preclinical evaluation of incretin-based therapeutic strategies
	Knockin INS-C94Y	Reduced β-cell mass and insulin level; dilation of the endoplasmic reticulum in β cell; cataract development	Preclinical testing of novel treatment strategies
Congenital heart disease [39]	Combine patient-specific human-induced pluripotent stem cell-derived cardiomyocytes with pig hearts’ extracellular-matrix hydrogel	Abnormal phenotype of the long QT syndrome and catecholaminergic polymorphic ventricular tachycardia; occurrence of reentrant arrhythmia	Study of inherited and acquired cardiac disorders and drug development and testing
Ischemia/reperfusion-induced myocardial infarction [40]	Knockout the Hippo pathway gene Salvador	Improved ejection fraction; reduced scar sizes; increased capillary density and reduced cardiomyocyte ploidy	Treating heart failure
Heart failure (HF) [41]	Knockout the small ubiquitin-related modifier 1 gene	Improved cardiac function and stabilized left ventricular volumes	An approach to human HF therapy

**Table 3:** Genetically engineered NHP models.

Disease	Genetic Modification	Phenotype	Potential Applications
Huntington’s disease (HD) [55]	Knockin polyglutamine-expanded human huntingtin (HTT) gene	Observed nuclear inclusions and neuropil aggregates; dystonia and chorea	Underlying biology of HD and the development of potential therapies
Parkinson’s disease (PD) [56]	Knockout PINK1 gene	Reduced neuronal cells in cortex and striatum; decreased gray matter density in the cortex	A tool to investigate the diverse functions of PINK1 and the pathogenesis related to PINK1 dysfunction
Duchenne muscular dystrophy (DMD) [57]	Snip out a section of the dystrophin gene	Loss of dystrophin	A tool to understand the pathogenesis of DMD
Rett Syndrome [58]	Knockin MECP2 gene	Observed increase in frequency of repetitive circular locomotion; increase in anxiety; reduced social interaction; relatively weak cognitive phenotypes	--

## Data Availability

Not applicable.
